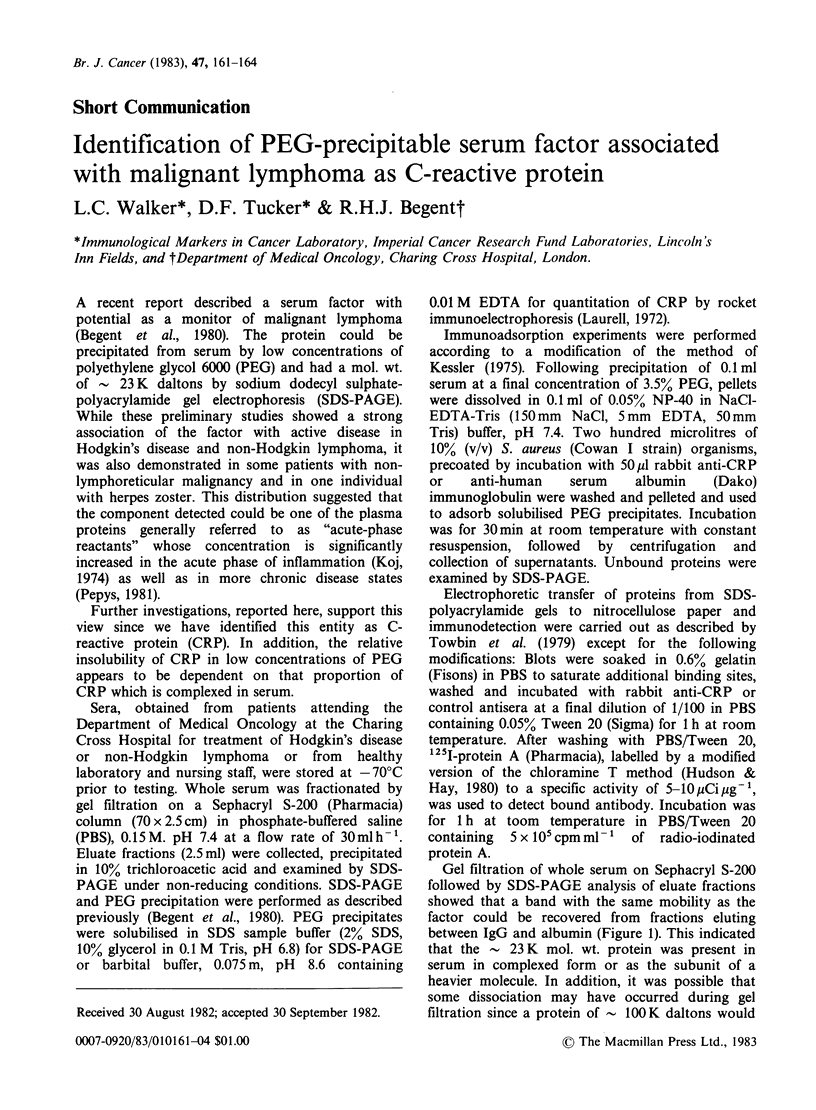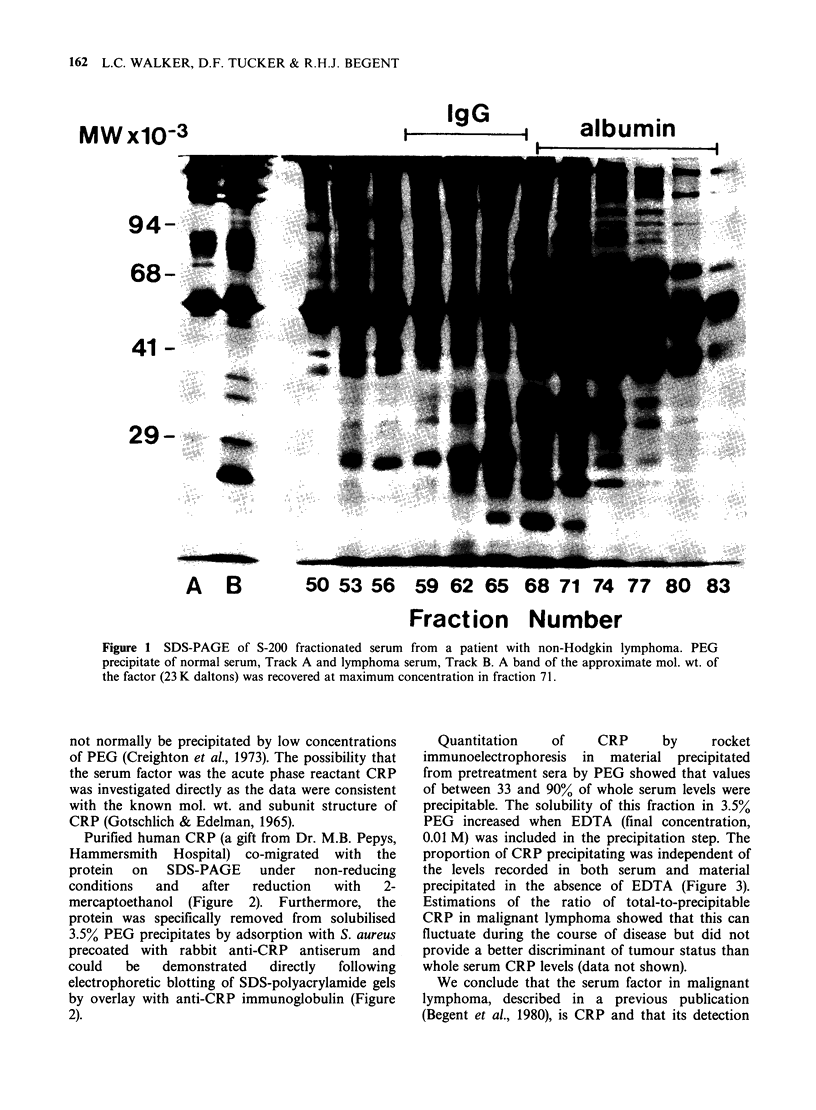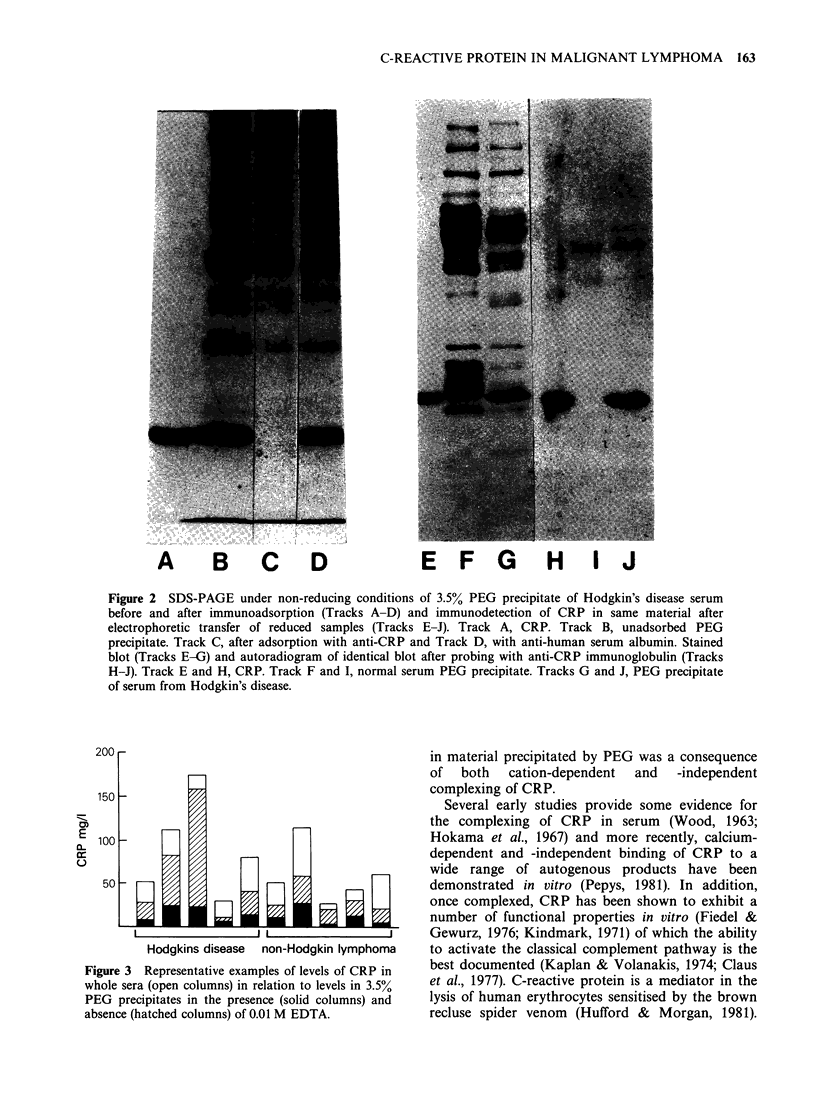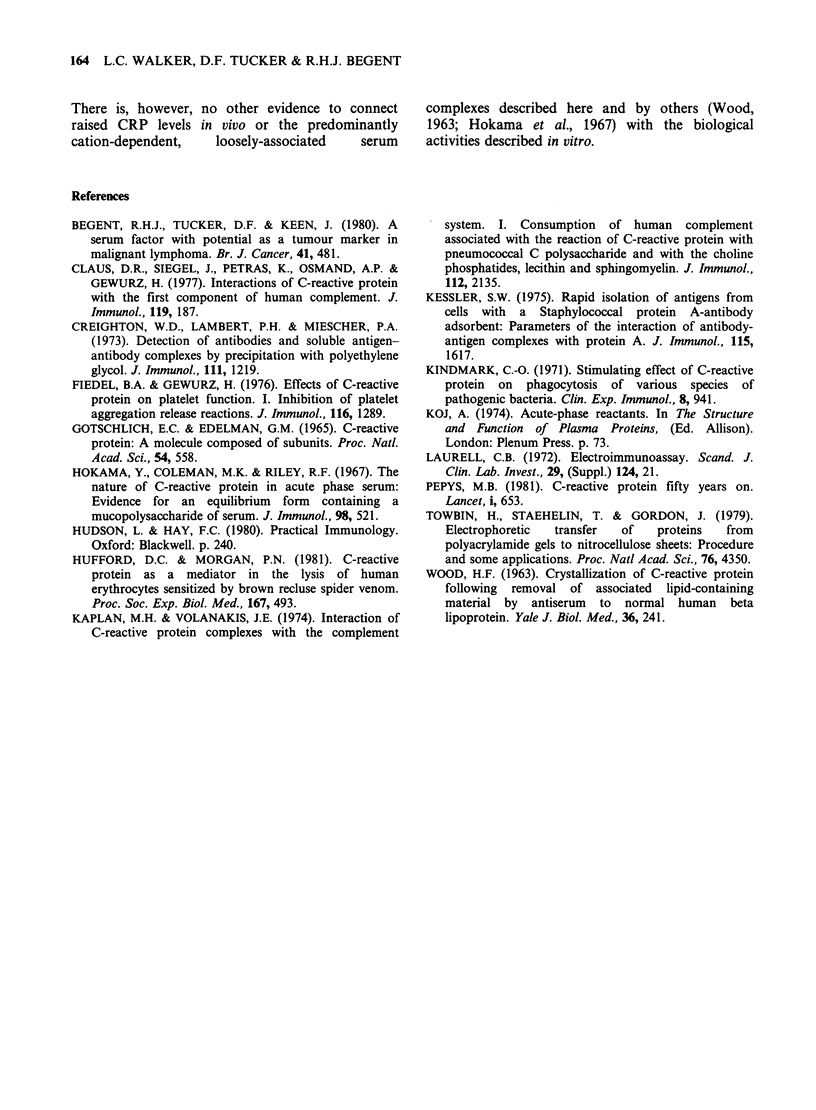# Identification of PEG-precipitable serum factor associated with malignant lymphoma as C-reactive protein.

**DOI:** 10.1038/bjc.1983.20

**Published:** 1983-01

**Authors:** L. C. Walker, D. F. Tucker, R. H. Begent

## Abstract

**Images:**


*Br. J. Cancer* (1983), **47**, 161–164

### Short Communication

### Identification of PEG-precipitable serum factor associated with malignant lymphoma as C-reactive protein

L.C. Walker*, D.F. Tucker* & R.H.J. Begent†

**Immunological Markers in Cancer Laboratory, Imperial Cancer Research Fund Laboratories, Lincoln's Inn Fields, and †Department of Medical Oncology, Charing Cross Hospital, London.*

A recent report described a serum factor with potential as a monitor of malignant lymphoma (Begent *et al.*, 1980). The protein could be precipitated from serum by low concentrations of polyethylene glycol 6000 (PEG) and had a mol. wt. of ~ 23K daltons by sodium dodecyl sulphate-polyacrylamide gel electrophoresis (SDS-PAGE). While these preliminary studies showed a strong association of the factor with active disease in Hodgkin's disease and non-Hodgkin lymphoma, it was also demonstrated in some patients with non-lymphoreticular malignancy and in one individual with herpes zoster. This distribution suggested that the component detected could be one of the plasma proteins generally referred to as "acute-phase reactants" whose concentration is significantly increased in the acute phase of inflammation (Koj, 1974) as well as in more chronic disease states (Pepys, 1981).

Further investigations, reported here, support this view since we have identified this entity as C-reactive protein (CRP). In addition, the relative insolubility of CRP in low concentrations of PEG appears to be dependent on that proportion of CRP which is complexed in serum.

Sera, obtained from patients attending the Department of Medical Oncology at the Charing Cross Hospital for treatment of Hodgkin's disease or non-Hodgkin lymphoma or from healthy laboratory and nursing staff, were stored at −70°C prior to testing. Whole serum was fractionated by gel filtration on a Sephacryl S-200 (Pharmacia) column (70 × 2.5 cm) in phosphate-buffered saline (PBS), 0.15 M. pH 7.4 at a flow rate of $30\,ml\,h^{-1}$. Eluate fractions (2.5 ml) were collected, precipitated in 10% trichloroacetic acid and examined by SDS-PAGE under non-reducing conditions. SDS-PAGE and PEG precipitation were performed as described previously (Begent *et al.*, 1980). PEG precipitates were solubilised in SDS sample buffer (2% SDS, 10% glycerol in 0.1 M Tris, pH 6.8) for SDS-PAGE or barbital buffer, 0.075 m, pH 8.6 containing 0.01 M EDTA for quantitation of CRP by rocket immunoelectrophoresis (Laurell, 1972).

Immunoadsorption experiments were performed according to a modification of the method of Kessler (1975). Following precipitation of 0.1 ml serum at a final concentration of 3.5% PEG, pellets were dissolved in 0.1 ml of 0.05% NP-40 in NaCl-EDTA-Tris (150 mm NaCl, 5 mm EDTA, 50 mm Tris) buffer, pH 7.4. Two hundred microlitres of 10% (v/v) *S. aureus* (Cowan I strain) organisms, precoated by incubation with 50 μl rabbit anti-CRP or anti-human serum albumin (Dako) immunoglobulin were washed and pelleted and used to adsorb solubilised PEG precipitates. Incubation was for 30 min at room temperature with constant resuspension, followed by centrifugation and collection of supernatants. Unbound proteins were examined by SDS-PAGE.

Electrophoretic transfer of proteins from SDS-polyacrylamide gels to nitrocellulose paper and immunodetection were carried out as described by Towbin *et al.* (1979) except for the following modifications: Blots were soaked in 0.6% gelatin (Fisons) in PBS to saturate additional binding sites, washed and incubated with rabbit anti-CRP or control antisera at a final dilution of 1/100 in PBS containing 0.05% Tween 20 (Sigma) for 1 h at room temperature. After washing with PBS/Tween 20, $^{125}I$-protein A (Pharmacia), labelled by a modified version of the chloramine T method (Hudson & Hay, 1980) to a specific activity of $5–10\,\mu Ci\,\mu g^{-1}$, was used to detect bound antibody. Incubation was for 1 h at toom temperature in PBS/Tween 20 containing $5 \times 10^5\,cpm\,ml^{-1}$ of radio-iodinated protein A.

Gel filtration of whole serum on Sephacryl S-200 followed by SDS-PAGE analysis of eluate fractions showed that a band with the same mobility as the factor could be recovered from fractions eluting between IgG and albumin (Figure 1). This indicated that the ~ 23K mol. wt. protein was present in serum in complexed form or as the subunit of a heavier molecule. In addition, it was possible that some dissociation may have occurred during gel filtration since a protein of ~ 100K daltons would

Received 30 August 1982; accepted 30 September 1982.

0007-0920/83/010161–04 $01.00

© The Macmillan Press Ltd., 1983

**Figure 1** SDS-PAGE of S-200 fractionated serum from a patient with non-Hodgkin lymphoma. PEG precipitate of normal serum, Track A and lymphoma serum, Track B. A band of the approximate mol. wt. of the factor (23 K daltons) was recovered at maximum concentration in fraction 71.

not normally be precipitated by low concentrations of PEG (Creighton *et al.*, 1973). The possibility that the serum factor was the acute phase reactant CRP was investigated directly as the data were consistent with the known mol. wt. and subunit structure of CRP (Gotschlich & Edelman, 1965).

Purified human CRP (a gift from Dr. M.B. Pepys, Hammersmith Hospital) co-migrated with the protein on SDS-PAGE under non-reducing conditions and after reduction with 2-mercaptoethanol (Figure 2). Furthermore, the protein was specifically removed from solubilised 3.5% PEG precipitates by adsorption with *S. aureus* precoated with rabbit anti-CRP antiserum and could be demonstrated directly following electrophoretic blotting of SDS-polyacrylamide gels by overlay with anti-CRP immunoglobulin (Figure 2).

Quantitation of CRP by rocket immunoelectrophoresis in material precipitated from pretreatment sera by PEG showed that values of between 33 and 90% of whole serum levels were precipitable. The solubility of this fraction in 3.5% PEG increased when EDTA (final concentration, 0.01 M) was included in the precipitation step. The proportion of CRP precipitating was independent of the levels recorded in both serum and material precipitated in the absence of EDTA (Figure 3). Estimations of the ratio of total-to-precipitable CRP in malignant lymphoma showed that this can fluctuate during the course of disease but did not provide a better discriminant of tumour status than whole serum CRP levels (data not shown).

We conclude that the serum factor in malignant lymphoma, described in a previous publication (Begent *et al.*, 1980), is CRP and that its detection

**Figure 2** SDS-PAGE under non-reducing conditions of 3.5% PEG precipitate of Hodgkin's disease serum before and after immunoadsorption (Tracks A–D) and immunodetection of CRP in same material after electrophoretic transfer of reduced samples (Tracks E–J). Track A, CRP. Track B, unadsorbed PEG precipitate. Track C, after adsorption with anti-CRP and Track D, with anti-human serum albumin. Stained blot (Tracks E–G) and autoradiogram of identical blot after probing with anti-CRP immunoglobulin (Tracks H–J). Track E and H, CRP. Track F and I, normal serum PEG precipitate. Tracks G and J, PEG precipitate of serum from Hodgkin's disease.

**Figure 3** Representative examples of levels of CRP in whole sera (open columns) in relation to levels in 3.5% PEG precipitates in the presence (solid columns) and absence (hatched columns) of 0.01 M EDTA.

in material precipitated by PEG was a consequence of both cation-dependent and -independent complexing of CRP.

Several early studies provide some evidence for the complexing of CRP in serum (Wood, 1963; Hokama *et al.*, 1967) and more recently, calcium-dependent and -independent binding of CRP to a wide range of autogenous products have been demonstrated *in vitro* (Pepys, 1981). In addition, once complexed, CRP has been shown to exhibit a number of functional properties *in vitro* (Fiedel & Gewurz, 1976; Kindmark, 1971) of which the ability to activate the classical complement pathway is the best documented (Kaplan & Volanakis, 1974; Claus *et al.*, 1977). C-reactive protein is a mediator in the lysis of human erythrocytes sensitised by the brown recluse spider venom (Hufford & Morgan, 1981).

There is, however, no other evidence to connect raised CRP levels *in vivo* or the predominantly cation-dependent, loosely-associated serum complexes described here and by others (Wood, 1963; Hokama *et al.*, 1967) with the biological activities described *in vitro*.

### References

BEGENT, R.H.J., TUCKER, D.F. & KEEN, J. (1980). A serum factor with potential as a tumour marker in malignant lymphoma. *Br. J. Cancer,* **41,** 481.

CLAUS, D.R., SIEGEL, J., PETRAS, K., OSMAND, A.P. & GEWURZ, H. (1977). Interactions of C-reactive protein with the first component of human complement. *J. Immunol.,* **119,** 187.

CREIGHTON, W.D., LAMBERT, P.H. & MIESCHER, P.A. (1973). Detection of antibodies and soluble antigen–antibody complexes by precipitation with polyethylene glycol. *J. Immunol.,* **111,** 1219.

FIEDEL, B.A. & GEWURZ, H. (1976). Effects of C-reactive protein on platelet function. I. Inhibition of platelet aggregation release reactions. *J. Immunol.,* **116,** 1289.

GOTSCHLICH, E.C. & EDELMAN, G.M. (1965). C-reactive protein: A molecule composed of subunits. *Proc. Natl. Acad. Sci.,* **54,** 558.

HOKAMA, Y., COLEMAN, M.K. & RILEY, R.F. (1967). The nature of C-reactive protein in acute phase serum: Evidence for an equilibrium form containing a mucopolysaccharide of serum. *J. Immunol.,* **98,** 521.

HUDSON, L. & HAY, F.C. (1980). Practical Immunology. Oxford: Blackwell. p. 240.

HUFFORD, D.C. & MORGAN, P.N. (1981). C-reactive protein as a mediator in the lysis of human erythrocytes sensitized by brown recluse spider venom. *Proc. Soc. Exp. Biol. Med.,* **167,** 493.

KAPLAN, M.H. & VOLANAKIS, J.E. (1974). Interaction of C-reactive protein complexes with the complement system. I. Consumption of human complement associated with the reaction of C-reactive protein with pneumococcal C polysaccharide and with the choline phosphatides, lecithin and sphingomyelin. *J. Immunol.,* **112,** 2135.

KESSLER, S.W. (1975). Rapid isolation of antigens from cells with a Staphylococcal protein A-antibody adsorbent: Parameters of the interaction of antibody-antigen complexes with protein A. *J. Immunol.,* **115,** 1617.

KINDMARK, C.-O. (1971). Stimulating effect of C-reactive protein on phagocytosis of various species of pathogenic bacteria. *Clin. Exp. Immunol.,* **8,** 941.

KOJ, A. (1974). Acute-phase reactants. In *The Structure and Function of Plasma Proteins,* (Ed. Allison). London: Plenum Press. p. 73.

LAURELL, C.B. (1972). Electroimmunoassay. *Scand. J. Clin. Lab. Invest.,* **29,** (Suppl.) **124,** 21.

PEPYS, M.B. (1981). C-reactive protein fifty years on. *Lancet,* **i,** 653.

TOWBIN, H., STAEHELIN, T. & GORDON, J. (1979). Electrophoretic transfer of proteins from polyacrylamide gels to nitrocellulose sheets: Procedure and some applications. *Proc. Natl Acad. Sci.,* **76,** 4350.

WOOD, H.F. (1963). Crystallization of C-reactive protein following removal of associated lipid-containing material by antiserum to normal human beta lipoprotein. *Yale J. Biol. Med.,* **36,** 241.